# Tissue-Specific Actions of Glucocorticoids on Apoptosis: A Double-Edged Sword

**DOI:** 10.3390/cells2020202

**Published:** 2013-03-26

**Authors:** Amanda L. Gruver-Yates, John A. Cidlowski

**Affiliations:** Laboratory of Signal Transduction, National Institutes of Environmental Health Sciences, 111 T. W. Alexander Drive, Research Triangle Park, NC 27709, USA; E-Mail: gruveral@niehs.nih.gov

**Keywords:** glucocorticoids, glucocorticoid receptors, apoptosis

## Abstract

First described for their metabolic and immunosuppressive effects, glucocorticoids are widely prescribed in clinical settings of inflammation. However, glucocorticoids are also potent inducers of apoptosis in many cell types and tissues. This review will focus on the established mechanisms of glucocorticoid-induced apoptosis and outline what is known about the apoptotic response in cells and tissues of the body after exposure to glucocorticoids. Glucocorticoid-induced apoptosis affects the skeletal system, muscular system, circulatory system, nervous system, endocrine system, reproductive system, and the immune system. Interestingly, several cell types have an anti-apoptotic response to glucocorticoids that is cytoprotective. Lastly, we will discuss the pro- and anti-apoptotic effects of glucocorticoids in cancers and their clinical implications.

## 1. Introduction

**Figure 1:**
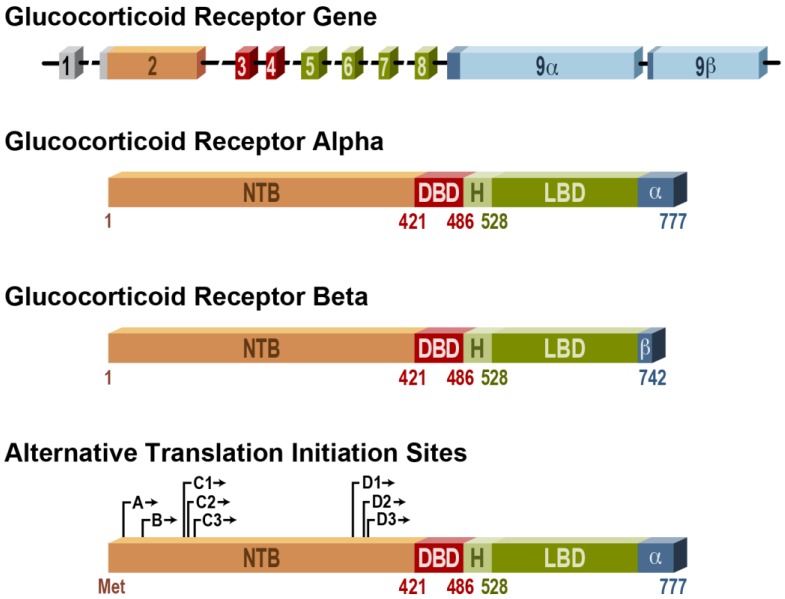
Glucocorticoid Receptor (GR) Gene Structure.

Glucocorticoids are essential for life [1], and their metabolic and immunosuppressive effects have been well established. Due to these potent anti-inflammatory effects, they are widely prescribed in clinical settings for a variety of medical conditions [2]. While essential for many therapies and treatments, an imbalance from stress or prolonged use in clinical applications may have overreaching or unintended consequences. Indeed, glucocorticoids are also potent inducers of apoptosis in many cell types. In glucocorticoid sensitive cells, apoptosis and other cellular effects are induced through the activation of the glucocorticoid receptor (GR). GR is a steroid hormone receptor that upon ligand binding translocates to the nucleus and exerts a myriad of genomic and non-genomic effects. The gene encoding the human GR (hGR) is on chromosome 5 in loci 31–32 (5q31–32) [2]. The receptor has three main functional domains; the *N*-terminal domain, the DNA-binding domain (DBD), and the ligand binding domain (LBD) (See Figure 1) [2]. Alternative splicing of exon 9 of the hGR results in two major isoforms: hGRα and hGRβ [3]. The highly expressed hGRα isoform is responsible for classical signaling and modulation of gene transcriptions, while the role of the hGRβ isoform is less defined and may function as a dominant negative inhibitor of hGRα signaling [3]. Furthermore, alternative translation initiation sites and posttranslational modifications of the GR result in various GR isoforms and a complex array of receptor molecules [2,4]. Interestingly, the different translational isoforms of GRα have been shown to induce apoptosis at different rates. The GRα-C isoform was identified as the most potent inducer of apoptosis, while the GRα-D isoform was the least potent inducer of apoptosis [5]. These data also suggest that the relative proportion of specific GR isoforms in tissues and cells may influence their response to GC-induced apoptosis [5].

**Figure 2:**
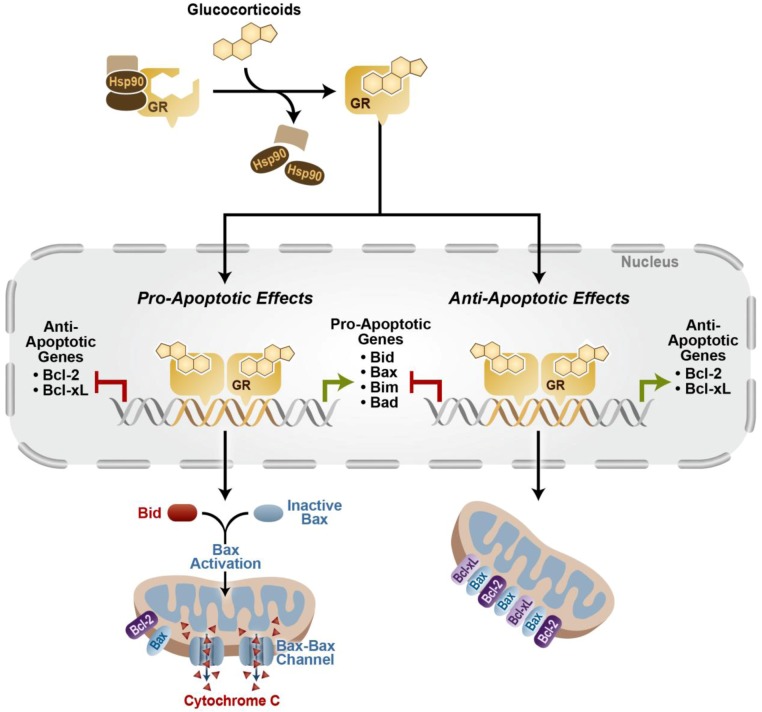
Glucocorticoids signal through GR to alter both pro- and anti-apoptotic genes that can either lead to apoptosis or cell survival depending on the cell type or tissue.

Several mechanisms are thought to contribute to glucocorticoid-induced apoptosis (GC-induced apoptosis). De novo transcription and translation events are necessary, since actinomycin D and cycloheximide block lymphocytes from depolarizing the plasma membrane and undergoing GC-induced apoptosis [6]. Additionally, GR DNA binding mutants that lack transactivation activity do not undergo GC-induced apoptosis [7]. While there is some evidence that glucocorticoids may act through the extrinsic apoptotic pathway, the classic mechanism of GC-induced apoptosis involves the activation of the intrinsic apoptotic pathway [4]. The extrinsic apoptotic pathway is characterized by the involvement of extrinsic signals (e.g., FasL, TRAIL) activating death receptors of the tumor necrosis factor (TNF) receptor superfamily, forming the Death Inducing Signaling Complex (DISC) that will activate caspase-8 to initiate apoptosis via either a mitochondrial-dependent or mitochondrial-independent mechanism [8]. The intrinsic apoptotic pathway involves the mitochondria and occurs in response to various intrinsic stimuli (e.g., glucocorticoids, UV exposure, and starvation). Glucocorticoid signaling increases the expression of the pro-apoptotic Bcl-2 family member Bim, which can activate the pro-apoptotic proteins Bax/Bak to disrupt mitochondrial membrane potential, resulting in the release of cytochrome c and other apoptogenic proteins [9]. This leads to caspase 9 activation and subsequent effector caspase 3 activation and apoptosis [4,9]. Other factors that may influence the intrinsic pathway during GC-induced apoptosis include up-regulation of other pro-apoptotic proteins such as Bad and Puma, or down regulation of anti-apoptotic proteins such as Bcl-2 or Bcl-xL [8]. This balance of the pro- and anti-apoptotic proteins can be altered by glucocorticoids, and whether a pro-apoptotic or anti-apoptotic effect is induced is often tissue- and/or cell type-specific (See Figure 2).

## 2. GC-Induced Apoptosis in Organ Systems

### 2.1. Skeletal System

GC-induced osteoporosis is a serious and prevalent side-effect of many medical treatments [10]. Glucocorticoids have anti-proliferative and pro-apoptotic effects on osteoblasts, the cells responsible for bone formation [10]. Known mechanisms of action include up-regulation of pro-apoptotic proteins such as Bim, and down-regulation of anti-apoptotic proteins, such as Bcl-2, Bcl-XL, and Mcl-1 [10]. Recently, dexamethasone was also found to inhibit murine osteoblast MC3T3-E1 cell growth by inducing G1 phase arrest and apoptosis through a p53-dependent up-regulation of the CDK inhibitor p21, and by up-regulation of the pro-apoptotic proteins NOXA and PUMA [11]. In contrast, glucocorticoid effects in osteoclasts, the cells responsible for bone resorption, are more ambiguous. Glucocorticoids may influence osteoclastogenesis by modulating the profile of cytokines produced by osteoblasts [10]. It is also unclear if osteoclasts express GR and whether or not GR or glucocorticoids directly regulate osteoclastogenesis [10]. Interestingly, glucocorticoids have been reported to increase osteoclast survival, as demonstrated both in cell culture and in mice [10]. Overall, GC-induced apoptosis in osteoblasts, the cells responsible for bone formation, and possible increased survival in osteoclasts, the cells responsible for bone resorption, can have a negative impact on bone health in response to excess glucocorticoids.

Additionally, glucocorticoids have been shown to exert apoptotic effects on cells of the cartilage, or chondrocytes. Dexamethasone treatment induced apoptosis in chondrocytes and contributed to the impairment of bone growth in mice [12]. A critical role for the pro-apoptotic protein Bax was identified in chondrocytes, and removal of the Bax protein was able to protect against dexamethasone-induced chondrocyte apoptosis in both cell lines and mice [12]. Furthermore, it is well-known that glucocorticoid treatment also inhibits chondrocyte proliferation, hypertrophy, and cartilage matrix production, contributing to decreased longitudinal bone growth [13]. Dexamethasone was found to induce apoptosis in a chondrocyte cell line by activating caspases and inhibiting the PI3K/AKT pathway [14]. These data show that excess glucocorticoids, either from acute exposure or prolonged clinical treatments, can stimulate a pro-apoptotic effect which can have a negative impact on the skeletal system and likely contributes to osteoporosis.

### 2.2. Skeletal Muscular System

An excess of glucocorticoids can lead to the development of myopathy, a disease resulting in muscle weakness [15]. Mechanisms of GC-induced myopathy include increased protein catabolism [16] and GC-induced apoptosis. Evidence exists for several mechanisms of action regarding GC-induced apoptosis in muscle cells, including mitochondrial-mediated apoptosis, Fas-mediated apoptosis, involvement of the proteasome, suppression of IGF-I signaling and the role of ceramide in glucocorticoid-mediated apoptosis [15]. Despite the prevalence of GC-induced myopathy following corticosteroid therapies, few studies have addressed the direct mechanisms of apoptosis in muscle cells, with much being derived from studies done on other cell types, particularly thymocytes. What is known, however, is that several synthetic glucocorticoids can induce apoptosis in a variety of muscle cells. The synthetic corticosteroid triamcinolone acetonide (TA) was found to induce apoptosis in soleus muscle of rats as shown by *in situ* end labeling (ISEL) and electron microscopy [17]. The authors found that the GC-induced apoptosis of skeletal muscle in TA-induced myopathy involved Fas-Fas ligand signals and the pro-apoptotic molecules of the extrinsic pathway, FADD and caspase 8 [17]. Fas expression, the pro-apoptotic proteins FADD, Bax, Bad, and Bid, and caspase 8 were all significantly increased in muscle fibers in response to TA treatment [17]. Corticosterone treatment in a human rhabdomyosarcoma cell line (solid tumor of muscle origin), has been shown to induce apoptosis primarily through ROS generation, which may contribute to steroid myopathy [18]. Dexamethasone treatment in rat L6 muscle cells also induced apoptosis, and similarly is thought to be linked to a ROS-generating mechanism [19]. An interesting model to study muscular disease is the mdx mouse model, in which a spontaneous mutation in the X-linked dystrophin gene results in muscle weakness [20]. Prednisolone treatment of mdx mice induced cell death in tibial anterior and quadriceps muscles as shown by TUNEL staining [21]. Thus, this evidence shows strong apoptotic effects induced by glucocorticoids in muscle may directly contribute to muscle wasting seen with prolonged steroid use.

### 2.3. Respiratory System

Glucocorticoids also induce apoptosis in other types of muscle, such as smooth muscle. In the respiratory system, glucocorticoids can induce apoptosis in airway smooth muscle cells (ASMC), an effect documented in dexamethasone-treated rats [22]. GC-induced apoptosis in these ASMC cells was reported to be mediated through an increase in Bax expression and decrease in Bcl-2 expression [22]. Additionally, corticosteroids induced apoptosis in airway epithelium, which could contribute to persistent epithelium damage and asthma [23]. Little is known about the effect of low dose glucocorticoids on ASMC, although glucocorticoids are often included in the media of these cells growing *in vitro*, suggesting that the level of glucocorticoids is important. While there are many positive effects of glucocorticoids in the treatment of airway diseases, including the suppression of inflammation mediated by the immune system in asthma and the use of glucocorticoids in lung maturation in premature infants, more information is needed to evaluate how apoptosis affects both airway smooth muscle cells and epithelial cells and their role in the progression of asthmatic disease.

### 2.4. Circulatory System

There have been few studies looking at GC-induced apoptosis in non-cardiac cells of the circulatory system, and these have focused on endothelial cells that make up the vessels that distribute blood throughout the body. In one study, dexamethasone treatment in rats led to an increase in endothelial cell death and contributed to GC-induced hypertension [24]. Wistar rats that were injected with dexamethasone for five consecutive days experienced endothelial cell apoptosis that likely contributed to capillary structural rarefaction, which is associated with hypertension in animals and humans [24]. Furthermore, secondary damage to organs can occur through GC-induced endothelium apoptosis. Dexamethasone treatment in rats was found to selectively induce apoptosis in endothelial cells of the corpus luteum, resulting in ischemic necrosis throughout the tissue [25]. Despite these findings, the mechanism of a pro-apoptotic effect of systemic glucocorticoids *in vivo* on endothelial cell types is controversial. Conversely, several studies of endothelial cells *in vitro* have indicated an anti-apoptotic effect of endothelial cells. For example, cell lines of human umbilical vein endothelial origin are protected by glucocorticoids from various apoptotic stimuli [26,27]. Additional studies are needed to determine the direct and indirect mechanisms contributing to endothelial cell death and if GC-induced damage to endothelium may contribute to GC-induced cell death in other tissues.

### 2.5. Nervous System

The central nervous system is highly vascularized, and contains specialized cells called pericytes, which wrap around endothelial cells and support blood vessel homeostasis. Primary pericytes isolated and cultured from the central nervous system of rat micro vessels were found to exhibit dexamethasone-induced apoptosis, an effect that was antagonized by the GR antagonist RU486 [28]. Such an apoptotic effect may be an important step in vascular regression and clinical disease in the nervous system [28]. Other cell types in the nervous system that undergo apoptosis in response to glucocorticoids include cells of the eye. Prolonged or high doses of glucocorticoid treatment often can increase ocular pressure and changes in the trabecular meshwork cells (cells that drain the aqueous humor from the eye) that can lead to glaucoma [29]. Dexamethasone has been reported to induce apoptosis in bovine trabecular meshwork cells in culture, which may contribute to the progression of steroid-induced glaucoma [30]. Elevated doses of dexamethasone also induce apoptosis and necrosis in cultured bovine corneal epithelial cells [31] and cultured human corneal epithelial cells [32]. While the direct role of GC-induced apoptosis in the eye *in vivo* has yet to be elucidated, it is clear that critical eye cell types are sensitive to glucocorticoids.

Other nervous tissues that undergo GC-induced apoptosis include the brain, where high levels of circulating glucocorticoids can have an effect despite the presence of the blood-brain barrier. The synthetic glucocorticoid dexamethasone, but not the natural glucocorticoid corticosterone, can induce apoptosis in the hippocampus, specifically the dentate gyrus, of the rat brain, as shown by increased TUNEL staining of the granule cell layer [33]. Chronic high corticosterone can suppress neurogenesis in the hippocampus in rats, a mechanism that may be involved in depression [34]. The suppression of neurogenesis was mediated at least in part by reduced cell proliferation, although this study failed to directly address apoptosis [34]. However, several other laboratories have confirmed the sensitivity of mature neurons of the dentate gyrus specifically to dexamethasone-induced apoptosis [35,36,37]. Degeneration of the adult hippocampus may play a role in the progression of psychiatric disorders [38,39]. Interestingly, the apoptotic effects of the synthetic glucocorticoid dexamethasone in the dentate gyrus have been reported to be ameliorated by activation of the mineralocorticoid receptor (MR) through either aldosterone [37] or the natural glucocorticoid corticosterone [33]. Moreover, removal of corticosteroids by adrenalectomy can lead to hippocampal granule cell apoptosis, an effect that is thought to be mediated through loss of MR stimulation [40]. As a result, it is clear excessive increased or decreased GC stimulation can be toxic in the hippocampus. Recently, it has been shown that neonatal exposure to dexamethasone induces apoptosis of neural precursor cells (NPC) of the hippocampus and reduces the number of available for the generation of new neurons [41]. Other areas affected by glucocorticoids include the external granule layer of the developing cerebellum, where glucocorticoids also induce apoptosis in NPCs, resulting in permanent decreases in the number of cerebellar neurons in neonatal mice [42]. Natural glucocorticoids are necessary for regulating the development of the external granule layer, which eventually disappears naturally after neurogenesis is no longer needed [43]. Taken together, these implications are clinically relevant, since dexamethasone is routinely used in obstetrics and neonatal medicine, especially for mothers at risk of preterm delivery to reduce the frequency of respiratory complications that can lead to perinatal death [44]. For example, a study comparing the growth patterns in the brains of preterm neonatal infants treated with hydrocortisone or dexamethasone showed impaired cerebellar, but not cerebral, growth [45]. Clearly, both the loss of glucocorticoids and excess glucocorticoids are potentially toxic to the brain, and our understanding of glucocorticoid action on brain function and development still remains to be fully elucidated.

### 2.6. Digestive System

Organs of the digestive system include the mouth, esophagus, stomach, small intestine, pancreas, liver, gallbladder, and colon. Most of the literature of GC-induced apoptosis in the digestive system focuses on epithelial cell types. Rat gastric epithelial cells were found to undergo apoptosis by dexamethasone treatment [46]. Similarly, gastric epithelial cell cultures of human origin were also sensitive to hydrocortisone-induced apoptosis [47]. While it is unknown if GC-induced apoptosis of the gastric mucosa contributes to ulcer development, it is known that dexamethasone contributes to ulcer susceptibility by inhibiting prostaglandin synthetase and peroxidase [48].

### 2.7. Endocrine System

Glucocorticoids are also important in the endocrine system. Excess glucocorticoids often result in altered glucose metabolism that can contribute to the development of type II diabetes [49]. The pancreas, which produces insulin, is highly susceptible to the apoptotic effects of glucocorticoids. Both murine β-cells and INS-1 cells in culture have been shown to undergo dexamethasone-induced apoptosis [50]. Mechanisms of action focus on repression of the anti-apoptotic protein Bcl-2 and activation of calcineurin, leading to de-phosphorylation of the pro-apoptotic protein BAD and mitochondrial depolarization [50]. These apoptotic effects may contribute to impaired β-cell function that leads to the development of diabetes mellitus. Recently, the thioredoxin-interacting protein (TXNIP), a redox regulating protein, has been shown to be a novel mediator in β-cell death [51]. TXNIP was found to be up-regulated by dexamethasone in both murine and human beta islet cells, an effect that was blocked by the GR antagonist RU486 [51]. It was also shown that down-regulation of TXNIP could attenuate dexamethasone-induced apoptosis [51]. The extent of GC-induced apoptosis in disease progression *in vivo*, *versus* other mechanisms of gene regulation controlling insulin secretion, remains to be elucidated. Interestingly, dexamethasone treatment in rats can alter apoptotic gene expression by increasing expression of the pro-apoptotic gene Bax and decreasing expression of the anti-apoptotic gene Bcl-2 [52], suggesting a potential role for GC-induced apoptosis in the development of diabetes following prolonged glucocorticoid treatment.

### 2.8. Reproductive System

Little is known in the literature of the direct induction of apoptosis in cells of the reproductive system, despite glucocorticoids being important for reproduction. In rats, dexamethasone treatment *in vivo* can induce apoptosis in the placenta, which may contribute to pregnancy complications such as fetal growth retardation [53]. Interestingly, the effect of synthetic glucocorticoids on the female reproductive system is not limited to the primary patient, but can also extend to a female fetus. Recent investigation into the effects of fetal exposure to dexamethasone suggests there is impairment of human fetal oogenesis through an apoptotic mechanism, highlighting the need to investigate female fertility after fetal exposure to dexamethasone [54].

Similarly, few studies have been reported looking at GC-induced apoptosis in the male reproductive system. Some studies indicate that male mice treated with dexamethasone exhibit increased apoptosis in testicular germ cells [55,56,57]. Other studies have also shown that high levels of natural glucocorticoids can induce apoptosis in rat Leydig cells, which are the primary source of testosterone [58,59,60]. Mechanisms important for GC-induced Leydig cell apoptosis include Fas/FasL, activation of caspase-3, mitochondrial depolarization, and increased ROS generation [59]. Clearly, glucocorticoids can induce apoptosis in cells of the reproductive system, although more studies are needed to broaden our current understanding of glucocorticoid function in both the male and female reproductive system.

### 2.9. Immune System

The apoptotic effects of glucocorticoids in cells of the immune system have been well-studied over the years, and glucocorticoids are known to exhibit many pleiotropic effects [61,62,63]. Physiological GC-induced apoptosis plays an important role in the development and function of the immune system [64,65]. Glucocorticoids are important for T cell selection, immune system homeostasis, and resolution of the immune response following clearance of infection [64,65]. The anti-inflammatory and immune-modulatory effects of glucocorticoids and GC-induced apoptosis have led to the use of steroid therapy as an invaluable part of treatment in many clinical settings. High doses of glucocorticoids are well-known to induce apoptosis in thymocytes [66,67], T cells [68], B cells [69], macrophages [70], mature but not immature dendritic cells [71], eosinophils [72], and natural killer cells [73]. Interestingly, glucocorticoids have the opposite effect in neutrophils and actually protect these cells from apoptosis [72,74].

While many of the known effects and specific mechanisms of GC-induced apoptosis have been extensively covered in the literature [61,62,63], we will highlight here some of the more recent insights into the molecular mechanisms of action of GC-induced apoptosis in the immune system. A critical role for a novel factor, tumor necrosis factor alpha-induced protein 8 (TNFAIP8) in GC-induced thymocyte apoptosis was recently identified, and down-regulating TNFAIP8 was able to protect against dexamethasone-induced apoptosis in thymocytes [75]. The function of TNFAIP8 is currently undefined, although it does contain a death effector domain (DED) [76]. Furthermore, a role for microRNA bioprocessing was discovered to play an important role in GC-induced apoptosis in lymphocytes [77]. Dexamethasone was found to reduce the expression of important nuclear (Drosha and DGCR8/Pasha) and cytoplasmic (Dicer) microRNA processing enzymes, which enhances GC-induced apoptosis [77]. Overexpression of these microRNAs blunted GC-induced apoptosis [77]. These provide further insight into the mechanism of GC-induced lymphocyte apoptosis.

In addition to the nuclear translocation of GR and its downstream effects on apoptosis, mitochondrial translocation has been proposed as a mechanism of action in CD4+CD8+ double positive (DP) thymocytes, the thymocyte subset that makes up the majority of developing T cells in a healthy thymus [78]. Dexamethasone treatment was found to induce co-localization of the glucocorticoid receptor with the mitochondrial dye CMX-Ros within 30 minutes in this thymocyte subtype as shown by confocal microscopy [78]. Subcellular fractionation confirmed these results. Mitochondrial localization of hormone-bound glucocorticoid receptors may contribute to the unique sensitivity of CD4+CD8+ thymocytes despite their low levels of glucocorticoid receptor expression compared to other lymphoid cell types [78]. Overall, GC-induced apoptosis in immune cells has been well-studied and plays a major role in the anti-inflammatory aspects of clinical disease. Moreover, GC-induced apoptosis plays a major role in treating malignancies of the immune system, a topic discussed later in this review.

## 3. Anti-Apoptotic Effects of Glucocorticoid Signaling

### 3.1. Anti-Apoptotic Effects of Glucocorticoid Signaling in Normal Tissue

While glucocorticoids can induce apoptosis in a wide range of cell types throughout the body, many cell types exhibit an anti-apoptotic response to glucocorticoid signaling. We have already mentioned that neutrophils [72,74] exhibit an anti-apoptotic effect in response to glucocorticoids. Similarly, ovarian follicular cells have an anti-apoptotic response to glucocorticoid signaling, where they are protected against apoptosis by a variety of pro-apoptotic stimuli (e.g., serum-starvation, TNF-α) [79,80]. More commonly, glucocorticoid exposure to cells of epithelial origin have been known to exhibit an anti-apoptotic effect. In 1995, the suppression of glucocorticoids was first found to be important in the reduction of mammary glands after lactation stopped [81]. Those studies demonstrated that dexamethasone treatment in mice inhibited apoptosis of murine mammary epithelial cells in post-lactating glands [81]. Moreover, glucocorticoids can also regulate apoptosis in specialized skin epithelial cells, or keratinocytes. In addition to suppression of wound healing, dexamethasone has been shown to protect against UV-mediated apoptosis in keratinocytes [82]. Furthermore, dexamethasone has also been found to inhibit IFN-gamma and IFN-gamma plus anti-Fas-induced apoptosis in lung epithelial cells [83]. These data support the concept that some cells of epithelial origin promote an anti-apoptotic effect in response to glucocorticoids.

In addition to epithelial cell types, hepatocytes and adipocytes have also been documented to exhibit an anti-apoptotic response to glucocorticoids. Primary liver cells undergo spontaneous apoptosis in culture, and dexamethasone has been shown to inhibit this process in both human and rat hepatocytes in a dose-dependent manner [84]. The anti-apoptotic proteins Bcl-2 and Bcl-xL were increased by dexamethasone treatment, and the pro-apoptotic expression of Bax and translocation of Bad was decreased by dexamethasone treatment in these hepatocytes [84]. It is interesting that glucocorticoids can down-regulate anti-apoptotic proteins or up-regulate anti-apoptotic proteins in a tissue-specific manner. Additionally, other studies have implicated the up-regulation of the anti-apoptotic cellular FLICE inhibitory protein (cFLIP) in the mechanism of glucocorticoid-mediated protection of apoptosis in hepatocytes [85]. Furthermore, dexamethasone has been found to inhibit TNF-α-induced apoptosis in human adipocytes and preadipocytes [86]. These observations may help explain why adipose tissue increases in obesity, despite increased inflammatory factors, such as TNF-α, that should trigger adipocyte apoptosis [86].

Two additional organs where glucocorticoids are cytoprotective are the heart and the kidney. Glucocorticoids can protect against cardiac injury from myocardial ischemia/reperfusion [87,88,89]. Glucocorticoids can also directly inhibit cardiomyocyte apoptosis. Studies done with H9C2 cells, a rat cardiomyocyte cell line, indicated that dexamethasone could block serum starvation-induced apoptosis alone, and block starvation-induced apoptosis in conjunction with TNF-α, a potent inducer of apoptosis in cardiomyocytes [90]. This effect was blocked by the GR antagonist RU486, and by RNAi knockdown of the glucocorticoid receptor, indicating that GR is required for these anti-apoptotic effects [90]. This data was consistent with previous studies in the literature that showed glucocorticoids to inhibit apoptosis in primary rat cardiomyocytes [91,92]. Similarly, glucocorticoids are known to have a protective effect in the kidney. Dexamethasone protects rat renal mesangial cells from stress-induced apoptosis by up-regulating sphingosine-1 phosphate (S1P) levels, which can stimulate cell proliferation and counteract apoptotic mechanisms [93]. A similar mechanism of action has been identified in human fibroblast cells [94]. Dexamethasone was found to have an anti-apoptotic effect in murine podocytes, a specialized cell in the kidney [95]. Mechanisms of action were found to include a dexamethasone-induced decrease in p53, increase in Bcl-xL, and inhibition of apoptosis-inducing factor (AIF) translocation [95]. Co-treatment of 10 mM dexamethasone was protective against both TNF- or LPS-induced apoptosis of bovine glomerular endothelial cells [96]. Dexamethasone was also found to be renoprotective against ischemia-reperfusion injury specifically through an anti-apoptotic effect on human renal proximal tubular cells [97]. These anti-apoptotic effects were found to be mediated via a GR-dependent, nongenomic signaling pathway involving activation of MEK-ERK1/2 [97]. Thus, it is quite clear that several cell types found in normal tissue of heart and kidney exhibit an anti-apoptotic effect in response to glucocorticoids, rather than undergoing GC-induced apoptosis. Furthermore, several cell types including many cells of epithelial origin do not undergo apoptosis in response to glucocorticoids, indicating that glucocorticoids can have a protective effect in a tissue-specific manner.

### 3.2. Resistance and Anti-Apoptotic Effects of Glucocorticoid Signaling in Cancer

Glucocorticoids are extremely important and a first line of defense in the treatment of hematopoietic malignancies [98,99]. Glucocorticoid therapy is used to treat acute lymphoblastic leukemia (ALL), chronic lymphoblastic leukemia (CLL), multiple myeloma (MM), Hodgkin’s Lymphoma, and Non-Hodgkin’s Lymphoma [4]. However, glucocorticoid therapy has been limited by the emergence of glucocorticoid resistance in malignant lymphocytes [4]. Some cancers, such as leukemia of myelogenous lineage, are innately resistant to glucocorticoid therapy, while others develop resistance after prolonged glucocorticoid therapy or after relapse [4]. Mechanisms contributing to glucocorticoid resistance include altered expression of glucocorticoid receptor isoforms, altered GR expression levels, GR mutations, dis-regulation of pro- or anti-apoptotic proteins, or altered interactions with different kinases [4]. While these mechanisms are not exclusive to cancer cells, resistance to glucocorticoids has been studied most in cancers that rely on glucocorticoid therapy for a significant part of the treatment regimen. Other types of cancer susceptible to glucocorticoid resistance include osteosarcoma and small-cell lung carcinoma [100]. Interestingly, the shortest GR translational isoform, GRα-D, does not mediate cell death in osteosarcoma cells [101]. In those experiments, osteosarcoma cells transfected only with the GRα-D isoform did not undergo apoptosis upon dexamethasone treatment, nor was there any dexamethasone-induced antagonism of NF-κB activity [101]. It would be interesting to see if there was a shift in the relative quantity of the GRα-D isoform in cancer cells that develop resistance. Overall, glucocorticoid therapies in many hematopoietic malignancies remain the first line of defense, and the development of glucocorticoid resistance is often correlated with a poor prognosis.

In addition to the development of glucocorticoid resistance in cancer therapy, anti-apoptotic effects of glucocorticoids introduce an important caveat in the therapeutic strategies for many cancers. Steroids such as dexamethasone are often used with chemotherapy and radiation treatments of patients to combat nausea and vomiting [102,103]. The therapeutic outcome of many solid tumors and cancers may be negatively impacted by glucocorticoid treatment depending on the type of cancer. Dexamethasone induces an anti-apoptotic effect in a variety of cell lines derived from breast cancer, brain cancer, cervical cancer, bone cell cancer, melanoma, and neuroblastoma [104]. The majority of these cell lines were protected against chemotherapy-induced apoptosis by dexamethasone. Furthermore, several urological cell lines and freshly isolated prostate tumor cells have been reported to exhibit an anti-apoptotic effect induced by dexamethasone treatment [105]. Dexamethasone was also found to inhibit apoptosis and promote cell proliferation in bladder cancer cells, yet appeared also to repress cell invasion and metastasis [106]. In C6 glioma cells, derived from brain cancer cells, dexamethasone was found to inhibit apoptosis induced by staurosporine, etoposide and thapsigargin by up-regulating Bcl-xL [107]. Bcl-xL-mediated anti-apoptotic effects induced by dexamethasone were also found in a human gastric cancer cell line [108]. Dexamethasone also inhibited apoptosis in several pancreatic cell lines cultured *in vitro*, and inhibited apoptosis of pancreatic carcinoma cells xenografted to mice *in vivo* [109]. Dexamethasone protects ovarian epithelial cell cancer lines against apoptosis by up-regulation of the caspase inhibitor cIAP2 [110]. Tumor necrosis factor-related apoptosis inducing ligand (TRAIL) is a member of the tumor necrosis factor (TNF) family including FasL, and TNF-α. TRAIL has been used to induce apoptosis in thyroid cancers since TRAIL receptors are broadly expressed on thyroid cancer cells [111]. In follicular undifferentiated thyroid (FRO) cancer cells, dexamethasone protects against TRAIL-mediated apoptosis [112]. This effect was found to be dependent on the glucocorticoid receptor and linked to the up-regulation of the anti-apoptotic protein Bcl-xL [112]. Unfortunately, there are many types of cancer cells that exhibit an anti-apoptotic response to glucocorticoids.

In some tissues where glucocorticoids are generally cytoprotective in normal cell types, such as hepatocytes and breast epithelial cells, there is also an anti-apoptotic effect of glucocorticoids in cancers derived from these tissues. Much of the evidence for GC-induced protection against apoptosis has been shown in cancers of the liver. In one study, dexamethasone protected against serum starvation-induced apoptosis in rat hepatoma cells [113]. Apoptosis in these cells involves an increase in NF-κB, an effect that is blocked by dexamethasone. Furthermore, suppression of NF-κB in these rat hepatoma cells allowed GC-induced apoptosis to occur, suggesting that dexamethasone treatment increased NF-κB levels and contributed to the anti-apoptotic effects in these cells [113]. Another study found dexamethasone to inhibit UV-C-induced apoptosis through up-regulation of the anti-apoptotic protein Bcl-xL in rate hepatoma cells [114]. Both serum starvation and UV-C trigger the intrinsic apoptotic pathway. Interestingly, dexamethasone does not protect rat hepatoma cells from apoptosis induced by the extrinsic pathway trigger Fas Ligand (FasL). However, dexamethasone was found to inhibit apoptosis in TGF-beta treated rat hepatoma cells, also likely by increasing Bcl-xL expression in these cells [115]. Several other studies have shown glucocorticoids to be protective in hepatoma carcinoma cells against other apoptotic stimuli [116,117]. Glucocorticoid treatment may also be particularly detrimental in breast cancer. In one study of breast cancer xenografts, dexamethasone pre-treatment significantly reduced paclitaxel-induced apoptosis [118]. The systemic pre-treatment of dexamethasone was found to up-regulated the anti-apoptotic gene MKP-1 while down regulating pro-apoptotic genes such as Bid and TRAIL in breast cancer tumor cells, an effect that persisted for weeks [118]. More recently, microarray analysis of dexamethasone-induced anti-apoptotic genes in breast cancer cells lines revealed MKP-1 and SGK-1 proteins to be of particular importance in GC-induced chemotherapy resistance in breast cancer, with glucocorticoid treatment up-regulating both of these genes [119]. Moreover, RNAi knock down of these genes abrogates the anti-apoptotic effects of glucocorticoids in breast cancer cells [119]. Overall, potential mechanisms of action of glucocorticoid signaling leading to anti-apoptotic events rather than cell death include activation of MKP-1, NF-κB, SGK-1, ACK, and WNT pathways [8]. Clearly, the balance between GC-induced pro- and anti-apoptotic events in specific cell types play an important role in cell death or survival, and the pros and cons of including glucocorticoids in cancer treatments should be investigated further.

## 4. Other Clinical Implications and Concluding Remarks

Many of the negative side effects of glucocorticoid therapy, such as cataracts, glaucoma, skin atrophy, hypertension, muscle wasting, diabetes mellitus, and thymus atrophy, can be attributed to the transactivation of the glucocorticoid receptor and subsequent gene induction [120]. This has led to research focusing on improved therapies for inflammatory diseases with selective glucocorticoid receptor modulators (SGRMs) or “dissociative” ligands that permit GR transrepression of pro-inflammatory genes but are suggested to have less transactivation activity [120]. The non-steroidal plant-derived Compound A (CpdA) has been proposed as a dissociative ligand of GR, and studies indicate it may have less side effects and be just as beneficial as dexamethasone to anti-inflammatory therapy in murine models of asthma [121], arthritis, [122] and experimental autoimmune encephalomyelitis [123]. Others have shown Compound A to selectively down-regulate the transcription factor T-bet in immune cells, while activating the transcription factor GATA-3, favoring a Th2 response over the inflammatory Th1 response of the immune system [124]. Perhaps this promotion of a less inflammatory Th2 response instead of a Th1 response may be the reason why CpdA has been promising in murine models of inflammation. Additional putative dissociative GR agonists that may be beneficial include ZK 216348, which has shown promise in murine models of skin inflammation [125], and mapracorat, which has shown promise in murine models of inflammation in the eye [126,127]. Because all the mechanisms of action elicited from SGRMs have yet to be elucidated, it is difficult to say how different these responses are to a normal glucocorticoid response, and to what degree they truly are “dissociative.” However, the need for selective modulators exists, and future research is needed, as these types of agents would have tremendous therapeutic value. It would also be very interesting to see what effects putative dissociative GR agonists would have on apoptotic pathways both in normal and cancer cells.

**Figure 3:**
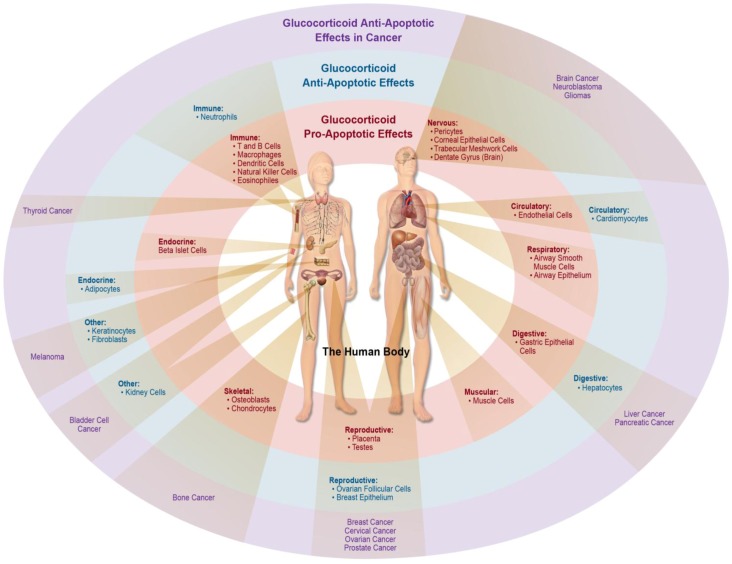
Tissue-specific effects of glucocorticoids on apoptosis in the human body.

The pro- or anti-apoptotic outcome of glucocorticoid signaling is highly dependent upon the cell type receiving the signal. Many cell types of the body undergo apoptosis in response to glucocorticoids (See Figure 3). However, there are many cell types that are resistant or respond in an anti-apoptotic manner to glucocorticoids (See Figure 3). Glucocorticoids are a widely prescribed class of drugs used to combat inflammation and disease, and are often included in many types of cancer treatments. Unfortunately, it seems many cancer cells are able to respond to glucocorticoid signaling in a way that promotes cell survival rather than induces apoptosis. Due to the far reaching and tissue- or cell-specific effects of glucocorticoids on the balance of pro- and anti-apoptotic responses, caution should be implemented in therapeutic strategies to ensure that we harness the great power of glucocorticoids while minimizing the risks.
